# The Clinical Significance of FilmArray Respiratory Panel in Diagnosing Community-Acquired Pneumonia

**DOI:** 10.1155/2017/7320859

**Published:** 2017-09-06

**Authors:** Huanzhu Chen, Huilan Weng, Meirui Lin, Ping He, Yazhen Li, Qingdong Xie, Changwen Ke, Xiaoyang Jiao

**Affiliations:** ^1^Cell Biology and Genetics Department, Shantou University Medical College, Shantou 515041, China; ^2^First Affiliated Hospital of Shantou University Medical College, Shantou 515041, China

## Abstract

**Aim:**

FilmArray Respiratory Panel (FilmArray RP) test is an emerging diagnostic method in fast detecting multiple respiratory pathogens; the methodology and clinical significance of FilmArray RP in community-acquired pneumonia (CAP) diagnosis were evaluated in this study.

**Methods:**

Specimens from 74 patients with CAP were analyzed and compared using FilmArray RP, traditional multiple PCR assay, bacterial (or fungal) culture, and serological detection.

**Results:**

FilmArray RP and multiplex PCR showed 100% coincidence rate in detecting coronaviruses 229E, OC43, HKU1, and NL63, human metapneumovirus, influenza A and B, and parainfluenza viruses (PIV1, PIV2, and PIV4). There were 15 viral specimens tested as disagreement positive results. FilmArray RP had higher detection rate in detecting dual viral and* Mycoplasma pneumoniae* infection. The positive bacteria (or fungi) were found in 25 specimens.

**Conclusions:**

This study demonstrated the capability of FilmArray RP for simultaneous detection of broad-spectrum respiratory pathogens and potential use in facilitating better patient care.

## 1. Introduction

The community-acquired pneumonia (CAP) is the leading cause of morbidity and mortality worldwide. According to WHO estimates, 450 million cases of pneumonia are recorded each year, with about 4 million deaths from this illness [1, 2], with the highest incidence cases in children younger than 5 years old [3]. The mortality rate has declined greatly due to early and accurate detection of the etiological agents, together with the timely initiation of appropriate treatment [4].

Nowadays, the golden standard for CAP diagnosis is still based on the chest radiography; however, a broad range of chest radiographic changes could be induced by various agents, a single and dual bacterium/virus, or mixed pathogens coinfections. These alterations of chest radiography are only helpful in specific cases to confirm a microbial cause of pneumonia [4]. Current diagnostic methods identify a pathogen in only 30%–40% of CAP patients [5, 6], and the frequent lack of a microbiological diagnosis in CAP impairs pathogen-directed antimicrobial therapy [7]. Polymerase chain reaction (PCR) technique has been shown to be reliable for diagnosing the pathogens, especially for those that are difficult to culture [4]. In addition, PCR method has high sensitivity and specificity in detecting multiple microorganisms and yields results faster than culture and serological methods [8]. Most importantly, the results of PCR are not affected by prior use of antibiotics. The findings indicate that the incidence of viral pneumonia in the past has been underestimated [4]. With multiple PCR assay, detection of multiple RNA or DNA targets in a single tube has the potential for rapid identification of complicated respiratory viral pathogens [9, 10]. The conventional techniques of microbial detection have high accuracy, but they are time- and labor-intensive, with limited range of detection and can be subjective, relying very much on technical expertise for the interpretation of cytopathic effect (CPE) in cell culture [11]. Direct fluorescent antibody assay (DFA) and immunochromatographic antigen testing, although rapid, have poor sensitivity for the detection of most viruses [12]. The serological methods also have low sensitivity. Therefore, an assay that is capable of rapid detection and accurate identification of multiple pathogens is desirable.

FilmArray Respiratory Panel (RP) is a multiplexed nucleic acid test for the simultaneous qualitative detection and identification of multiple respiratory virus,* Bordetella pertussis, Chlamydophila pneumoniae, and Mycoplasma pneumoniae*. Particularly, the whole process only takes about an hour; compared to multiple PCR, the FilmArray RP has provided fast results. As the FilmArray RP is emerging method, which is rarely employed in clinical specimen's detection, its clinical significance in diagnosing CAP is sparse. Therefore, in this study, we evaluated the capability of FilmArray RP for simultaneous identification of multiple pathogens; its microbial yield and clinical significance in CAP diagnosis were also evaluated.

## 2. Materials and Methods

### 2.1. Clinical Specimens

In all specimens of patients from the first and the second affiliated hospitals of Shantou University Medical College, CAP was confirmed by radiological diagnosis. The nasal cavity samples and serum were collected from patients between January and September, 2016.

### 2.2. Multiplex Real-Time PCR Assay

The specimens collected from nasal mucus were stored in swab storage solution (COPAN, Italy) for multiplex PCR and FilmArray RP assay. The nucleic acid was extracted by using beads viral DNA/RNA extraction kit (TIANLONG, China) and NP968 nucleic acid extraction instrument (TIANLONG, China). We tested 14 viruses, including influenza viruses types A and B (Flu A and Flu B), parainfluenza viruses 1 to 4 (PIV1–4), respiratory syncytial virus (RSV), coronaviruses 229E, OC43, HKU1, and NL63, human metapneumovirus (MPV), human rhinovirus (HRV), and adenovirus (ADV), were detected using a single-tube TaqMan® real-time reverse transcription PCR strategy (AgPath-ID™ One-Step RT-PCR Kit Applied Biosystems, USA). PCR amplification was performed using a 7500 Real-Time PCR System (Applied Biosystems, USA), according to the manufacturer's instructions.

### 2.3. Bacterial (or Fungi) Detection

The nasal mucus specimens were cultured according to China national standard protocols to detect respiratory bacterial (or fungi). Colony identification was undertaken using a VITEK 2 Compact system (Biomérieux, France). Nine bacteria were measured, including* Acinetobacter baumannii, Enterobacter aerogenes, Escherichia coli, Klebsiella ornithinolytica, Klebsiella pneumoniae, Pseudomonas aeruginosa, Staphylococcus aureus, Staphylococcus haemolyticus,* and* Bordetella pertussis*. One fungus (yeast) was also detected.

### 2.4. *Mycoplasma pneumoniae* and* Chlamydia pneumoniae* Detection

The patient's serum was collected and placed in −80°C.* Mycoplasma pneumoniae* and* Chlamydia pneumoniae* antibody (IgM) were detected by commercial kit (KANGHUA, China), in accordance with the instructions.

### 2.5. The FilmArray RP Assay

We used the BioFire™ Diagnostics Respiratory Panel and FilmArray™ multiplex PCR system (Biomérieux, USA) to detect the same 14 viruses and three other pathogens (*Bordetella pertussis, Chlamydophila pneumoniae,* and* Mycoplasma pneumoniae*) according to the package insert.

### 2.6. Statistical Analyses

Data were analyzed in SPSS 19.0. The agreement between assays was measured using the kappa statistic. The sensitivity and specificity were also compared for both tests.

## 3. Results

### 3.1. Patients' Biographic and Clinical Features

Patients were enrolled between January and September, 2016. This study was comprised of both adult (*n* = 37) and pediatric (*n* = 37) patients with female/male ratio of 0.68 : 1. Patients in this study could have received treatment with antibiotics (cephalosporins, astaxanthin, and sulbactam, resp.) and antiviral drugs (oseltamivir and ribavirin), in combination or alone. Four cases of patients had the other lung diseases, while 32 cases of patients had system diseases. Cough, fever, and shortness of breath or dyspnea were the most frequently symptoms observed in these patients. General characters are presented in Table 1.

### 3.2. Virus Detection in CAP Patients

The most prevalent were the RSV (15/74, 20.3%) and the HRV (9/74, 12.2%). Influenza viruses (A + B) were detected in 10 (10/74, 13.5%) samples. Coronaviruses (OC43, HKU1, and NL63) were detected in 9 (9/74, 12.2%) samples, PIV3 in 5 (5/74, 6.8%) samples, ADV in 3 (3/74, 4.1%) samples, and MPV in 2 (2/74, 2.7%) samples (Table 2).

### 3.3. Correlation between FilmArray RP and Conventional Multiplex PCR Assay

For viruses 229E, OC43, HKU1, NL63, MPV, Flu A, Flu B, PIV1, PIV2, and PIV4, two methods had very good agreement with coincidence rate of 100%. Disagreement was found in 15 specimens, positive results were tested by only one of the two assays (FilmArray RP: 12 cases, multiple PCR: 3 cases). FilmArray RP positive/PCR negative were seen in 3 cases of ADV, 6 cases of HRV, and 3 cases of RSV, while multiple PCR positive/FilmArray RP negative were observed in 1 case of PIV 3 and 2 cases of RSV (Table 2).

### 3.4. Sensitivity and Specificity of FilmArray RP in Detecting Virus

In the study, the FilmArray RP detected 50 positive viruses (ADV, 3; OC43, 7; HKU1, 1; NL63, 1; MPV, 2; HRV, 9; Flu A, 8; Flu B, 2; PIV3, 4; and RSV, 13, resp.). A total of 50 (100%) of the 50 pathogens were confirmed by real-time PCR; the comparison of FilmArray RP and multiple PCR for OC43, HKU1, NL63, MPV, Flu A, Flu B, PIV3, and RSV showed satisfactory agreement (kappa > 0.7) for all positive viruses except ADV and HRV (kappa = 0; kappa < 0.6). In total, FilmArray RP and multiple PCR had significantly high sensitivities and specificities for the detection of respiratory viruses, but compared to the multiple PCR, the sensitivity of FilmArray RP for PIV3 and RSV was different, with sensitivity of 80% and 83.3%, respectively (Table 2).

### 3.5. Dual Viral Infection Detected by FilmArray RP and Traditional Multiplex PCR Assay

Dual viral infections were detected in 12 (12/74, 16.2%) specimens (Table 3). Among them, 5 cases of dual viral infection were found in both methods, 6 cases were detected by FilmArray RP alone, and 1 case was detected by multiple PCR. In dual viral infection, majority was RSV combined with other respiratory viruses, a total of 9 cases, such as RSV + HRV positive (3 cases), RSV + ADV positive (2 cases), RSV + Flu A positive (1 case), RSV + HKU1 positive (1 case), RSV + NL63 positive (1 case), and RSV + PIV3 positive (1 case). The data indicated that FilmArray RP detected more viruses than multiple PCR, especially in dual viral infections. Intriguingly, the nondetected virus by PCR assay concentrated on RSV is accompanied by other respiratory virus.

### 3.6. Detection of Bacterial (or Fungi) or* Mycoplasma pneumoniae* Infections

Bacteria (or fungi) were detected by culture method in our study. The results showed that bacteria (or fungi) were positive in 25 (25/74, 33.8%) specimens. As FilmArray RP only detects* Bordetella pertussis*, it is hard to do method evaluation between two methods. In our study, 2 cases of* Bordetella pertussis* were detected by FilmArray RP. However, no* Bordetella pertussis* was isolated by culture method. The* Mycoplasma pneumoniae* found in 6 (6/74, 8.1%) specimens was only to be detected by FilmArray RP, while all serological tests to* Mycoplasma pneumoniae* were negative (Table 4).

## 4. Discussion

Microorganism measurement is an integral part of CAP patient management. Accurate and rapid etiological diagnosis helps prevent secondary infection, prevent the use of unnecessary antibiotics, facilitate more timely use of antiviral drugs, and shorten hospital stays [13]. In our study, even with bacterial (or fungal) culture, antibody IgM detection, multiple PCR, and FilmArray RP, there were 18/74 cases (24.32%) of CAP that could not find the pathogens.

The multiplex real-time PCR can detect a broad spectrum of respiratory microorganisms promptly, which makes it possible for clinician to treat the patients with specific antimicrobial agents. However, the main limitations of PCR method include the handling of whole batch samples (at least 8 samples) for cost and labor saving, the different boxes to do extraction, the training of specialized biologist that may be the main reason of DNA/RNA extraction and amplification being easily contaminated, and results interpretation (the frequency of false positive or false negative results hinders the use of PCR in clinical practice). Thus, the PCR is not appropriate for instant point of care especially to some severe patients. Compared with multiple PCR, the measurement time of FilmArray RP needs only about 1.2 hours and there is no need to do the experiment in batch. The single test set makes it meet the requirement of clinician to decide whether the patients should be quarantined or not. For some virus, such as Flu A, rapid diagnosis may help prevent secondary spread, beneficial for preventing virus nosocomial transmission. In children and adults, neuraminidase inhibitors reduce median time to resolution of symptoms by 0.5–2.5 days when administered within 48 h of onset of symptoms [14]. Early use of neuraminidase inhibitors reduces the development of complications such as pneumonia [15]. From these points, the FilmArray RP offers potential advantage in patient's treatment.

Due to undeveloped immune system, mixed infections with two or more respiratory viruses are common in children but are not easily detected by conventional methods; hence, the biological significance of dual infections currently is not well understood [16]. It will have more clinical significance to analyze the association between age and pathogens. In our study, FilmArray RP had higher sensitivity in detecting the dual viral infection than multiple PCR. Similarly, from previous study, RSV is the predominant virus inducing severe pneumonia in the population [17–19] and is found to be the most common cause of severe respiratory disease in infants [20]. RSA and Flu A continue to be the major causative agent in dual viral infection. In this study, the detection rate of rhinoviruses was higher than for other respiratory viruses, though its role in pneumonia is still questioned [21]. The prevalence of adenovirus-associated CAP is fairly low (3/74, 4.1%), which is similar to the previous study, but this type of infection is important to recognize because it might induce severe and fatal necrotizing pneumonia (especially serotypes 3, 7, and 14) [22]. For this, PCR is substantially more sensitive for identification of adenovirus than antigen detection [23]. Due to the wide range of pathogens responsible for CAP, in moderate or severe infection, broad-spectrum antimicrobial cover should be initiated before deescalating to narrow spectrum pathogen-directed agents once a microbiological diagnosis has been made [24, 25]. The ability to differentiate viral from bacterial pneumonia could have very important significance in patient's management, which may help clinicians treat patients more promptly and appropriately. In hospital, it is not uncommon of mix bacterial-viral coinfections. When bacterial culture identify positive, combining viral infection is frequently ignored by clinician. On the other hand, the decision to prescribe antibiotics may be influenced by the lag of diagnosis; especially in children, empirical antibiotic therapy is almost always initiated in these patients [26] because of their immunocompromised and concerns for severe disease. In previous study,* Mycoplasma pneumoniae* is a causative agent of community-acquired atypical pneumonia, and* Chlamydophila pneumoniae* is an obligate intracellular bacterium that causes acute respiratory infections and is a common cause of CAP [27]. We used serological method to detect* Mycoplasma pneumoniae* and* Chlamydia pneumoniae* rather than culture. To* Mycoplasma pneumoniae* and* Chlamydia pneumonia *detection, culture is highly specific but it is a complex process that has a long turnaround time, technically demanding, and offers limited sensitivity. Serology and PCR methods may provide a rapid diagnosis [28]. The limitations of serologic method include* Chlamydia pneumoniae* IgM or IgG reactivity might be caused by heterotypic antibodies. The test is evidently unable to discriminate between past and persistent infections [29]. Previously, weak positive results were frequently observed in* Mycoplasma pneumoniae* and* Chlamydia pneumonia *detection in our lab; the reason needs further clarification. Comparing the methods mentioned above, FilmArray RP has high specificity and sensitivity and could obtain result in a very short time.

For considering the cost of reagent, the FilmArray RP is more expensive than multiple PCRs; the reagent and consumable costs (in RMB) for the FilmArray RP and the multiple PCR were calculated to be $230 and $40 per patient, respectively. However, the FilmArray RP is essential for the administration of appropriate antiviral therapy, which dramatically shortens hands-on time (62.7 hours versus 1.2 hours); the reduction in average time to discontinuation of oseltamivir resulted in cost savings of approximately US$34.16 per patient [30]. In addition, FilmArray RP results in overall decreased duration of antibiotic use of half a day, a shorter stay (a quarter of a day less) in the hospital following admission and reduced time spent in isolation (a third of a day less) for patients who were viral positive after implementation of the FilmArray RP [12, 31]. Therefore, the clinical significance is far beyond its cost. Compared with multiple PCR, the FilmArray RP provides instant care testing results with a simplified enhanced workflow, compared with laboratory-developed PCR (LD-PCR) [32].

In the last decades, the emergence of severe acute respiratory syndrome (SARS), avian influenza A (H5N1) virus, and the 2009 pandemic influenza A (H1N1) virus has reemphasized the important role of respiratory viruses as causes of severe pneumonia [4]. Nowadays, newly discovered viruses, including bocavirus, PIV4, novel coronaviruses, and rhinoviruses, are increasingly being recognized as causes of respiratory illness [12], which lead to the extensive use of the FilmArray RP in detecting these novel viruses. One of limitations of the FilmArray RP is that it cannot be modified according to the needs of the clinicians and the laboratory, which could not add new viruses in the detection panel like the multiple PCR. Another limitation of the FilmArray RP is that it can only detect* Bordetella pertussis, Chlamydophila pneumoniae,* and* Mycoplasma pneumoniae*. Complicated bacterial (or fungal) cultures coexit in viral-bacterial (or fungi) infection; hence, from this point, bacterial (or fungal) cultures are essential for rational antibiotic selection and treatment success.

## Figures and Tables

**Table 1:** Patients' general characteristics.

	Cases
*Total*	74
*Age*	
0–14	37
≥15	37
*Gender*	
Male	44
Female	30
*Pathogen infection*	
Single virus	26
Single bacterial (or fungi)	10
Virus + bacterial (or fungi)	14
Single *Mycoplasma pneumoniae*	5
Virus + *Mycoplasma pneumoniae*	1
Unidentified	18
*Treatment*	
Antibiotic	45
Antiviral	7
Antiviral + antibiotic	11
*Other lung diseases*	
COPD	1
Tuberculosis	1
Asthma	2
*Systemic diseases*	
Diabetes	4
High blood pressure	14
Heart disease	3
Tumor	1
Hepatitis	6
Epilepsy	4
*Severity*	
Mild-moderate	47
Severity	27
*Clinical symptom*	
Cough	56
Pharyngeal discomfort	4
Snivel	10
Fever	42
Shortness of breath or dyspnea	25
*Prognosis after healing*	
Death	3
Alive	71

COPD, chronic obstructive pulmonary disease.

**Table 2:** Comparison of positive and negative results in two methods in viral detection.

Virus	Number of specimens (+: positive; −: negative)				Sensitivity (%)	Specificity (%)	PPV (%)	NPV (%)	Kappa
	PCR−/FA−	PCR+/FA+	PCR+/FA−	PCR−/FA+					
ADV	71	0	0	3	0	95.9	0	100	0
229E	74	0	0	0	0	100	0	100	1
OC43	67	7	0	0	100	100	100	100	1
HKU1	73	1	0	0	100	100	100	100	1
NL63	73	1	0	0	100	100	100	100	1
MPV	72	2	0	0	100	100	100	100	1
HRV	65	3	0	6	100	91.5	33.3	100	0.468
FluA	66	8	0	0	100	100	100	100	1
FluB	72	2	0	0	100	100	100	100	1
PIV1	74	0	0	0	0	100	0	100	1
PIV2	74	0	0	0	0	100	0	100	1
PIV3	69	4	1	0	80	100	100	98.6	0.882
PIV4	74	0	0	0	0	100	0	100	1
RSV	59	10	2	3	83.3	95.2	76.9	96.7	0.752

FA, FilmArray RP; PPV, positive predictive value; NPV, negative predictive value.

**Table 3:** The positive virus combination of FilmArray RP and multiplex real-time PCR in detecting dual viral infection.

	Multiplex PCR assay	FilmArray RP
Compositions	OC43 + HRV	OC43 + HRV
	Flu A + MPV	Flu A + MPV
	RSV + HKU1	RSV + HKU1
	RSV + PIV3	RSV + PIV3
	RSV + NL63	RSV + NL63
	RSV	RSV + *HRV*
	RSV	RSV + *HRV*
	RSV	RSV + *ADV*
	RSV	RSV + *ADV*
	Flu A	*RSV* + Flu A
	OC43	OC43 + *HRV*
	*RSV* + HRV	HRV

**Table 4:** The positive pathogens of bacterial (or fungi) or *Mycoplasma pneumoniae* detection and FilmArray RP in specimens detection.

Bacterial (or fungi) or *Mycoplasma pneumoniae* detection	FilmArray RP (—: not detected)	Cases
Yeast	—	6
Negative	*Mycoplasma pneumoniae*	6
*Staphylococcus aureus*	—	4
*Acinetobacter baumannii*	—	3
*Enterobacter aerogenes*	—	2
*Klebsiella pneumoniae*	—	2
*Pseudomonas aeruginosa*	—	2
*Escherichia coli *	*Bordetella pertussis*	1
*Escherichia coli *	—	1
*Klebsiella ornithinolytica*	—	1
*Negative*	*Bordetella pertussis*	1
*Staphylococcus haemolyticus*	—	1
